# Immunotherapy using anti-PD-1 and anti-PD-L1 in *Leishmania amazonensis*-infected BALB/c mice reduce parasite load

**DOI:** 10.1038/s41598-019-56336-8

**Published:** 2019-12-30

**Authors:** Alessandra M. da Fonseca-Martins, Tadeu D. Ramos, Juliana E. S. Pratti, Luan Firmino-Cruz, Daniel Claudio Oliveira Gomes, Lynn Soong, Elvira M. Saraiva, Herbert L. de Matos Guedes

**Affiliations:** 1 Instituto de Biofísica Carlos Chagas Filho, Laboratório de Imunofarmacologia, Grupo de Imunologia e Vacinologia, Universidade Federal do Rio de Janeiro, Rio de Janeiro, RJ Brazil; 2 Paulo de Góes Microbiology Institute, Immunology Department, Universidade Federal do Rio de Janeiro, Rio de Janeiro, RJ Brazil; 3 Universidade Federal do Rio de Janeiro – Campus Duque de Caxias Professor Geraldo Cidade, Rio de Janeiro, RJ Brazil; 4 Núcleo de Doenças Infecciosas, Universidade Federal do Espírito Santo, Vitória, ES Brazil; 5 Department of Microbiology and Immunology, Institute for Human Infections and Immunity, University of Texas Medical Branch, Galveston, TX USA; 6 Laboratório Interdisciplinar de pesquisas Médicas, Instituto Oswaldo Cruz, Fundação Oswaldo Cruz, Rio de Janeiro, Brazil

**Keywords:** Immunotherapy, Parasite immune evasion, Infection

## Abstract

Leishmaniasis is a neglected disease, for which current treatment presents numerous issues. *Leishmania amazonensis* is the etiological agent of cutaneous and diffuse cutaneous leishmaniasis. The roles of the programmed death-1 (PD-1) receptor on lymphocytes and its ligand (PD-L1) on antigen-presenting cells have been well studied in tumor and other infection models; but little is known about their roles in non-healing cutaneous leishmaniasis. In this study, we observed that *L. amazonensis* induced PD-1 expression on both CD4^+^ and CD8^+^ T cells and PD-L1 on dendritic cells on BALB/c mice. We tested the therapeutic potential of anti-PD-1 and anti-PD-L1 monoclonal antibodies (MoAbs) against a non-healing *L. amazonensis* infection in BALB/c mice, and that anti-PD-1 and anti-PD-L1 treatment significantly increased IFN-γ-producing CD4^+^ and CD8^+^ T cells, respectively. Compared with infection controls, mice treated with anti-PD-1 and anti-PD-L1, but not anti-PD-L2, displayed bigger lesions with significantly lower parasite loads. Treatment did not affect anti-*Leishmania* antibody (IgM, IgG, IgG1 and IgG2a) or IL-10 production, but anti-PD-1 treatment reduced both IL-4 and TGF-β production. Together, our results highlight the therapeutic potential of an anti-PD-1-based treatment in promoting the reinvigoration of T cells for the control of parasite burden.

## Introduction

Leishmaniasis is a disease with global public health concerns, particularly in poor communities. Conventional treatments arose in the 1940s; however, many problems are associated with these medications, such as high toxicity, adverse side effects, and the increased incidence of drug-resistant parasites. At present, there are no vaccines available for human use, which makes the search for new molecular targets highly necessary^1^. *Leishmania amazonensis* infection can cause a diverse spectrum of the disease, including cutaneous (the most common), mucosal, and visceral leishmaniasis, as well as diffuse cutaneous leishmaniasis that is refractory to the conventional treatment^2^.

The programmed death-ligand 1 (PD-L1), a cell surface glycoprotein belonging to the B7 family is expressed on antigen-presenting cells such as neutrophils, macrophages, and dendritic cells. PD-L1 binds to the PD-1 receptor, which belongs to the CD28 family and is expressed on T cells, B cells, and myeloid cells^3–5^. The PD-1/PD-L1 interaction leads to the suppression of T cells by affecting the gradual loss of cell activities including cytokine secretion (IFN-γ, IL-2, TNF-α), decreasing the proliferative capacity, and finally, inducing T cell apoptosis^6,7^. The PD-L1 receptor is widely discussed in oncological studies, as it is selectively expressed in many tumors^4,8,9^ and in cells within the tumor microenvironment in response to inflammatory stimuli^10^. PD-L1 is positively regulated in solid tumors, where it can inhibit cytokine production and the cytolytic activity of PD-1-expressing CD4^+^ and CD8^+^ T cells^4,11,12^. PD-1/PD-L1-based monoclonal antibody (MoAb) therapy is currently in phase III clinical trials with promising results for treatment against bladder carcinoma^13^ and lung cancer^14^. Programmed death-ligand 2 (PD-L2) is also a cell surface glycoprotein in the B7 family and plays a role similar to PD-L1, because it inhibits T cell function by binding PD-1 to the controversy in different models. T cell suppression is also reversed when the receptor is blocked by a specific antibody, for example, in inducing oral tolerance^15–17^.

It has been shown that PD-1/PD-L1-mediated cellular exhaustion also occurs during the progression of chronic infectious diseases caused by viruses or protozoan parasites, such as AIDS, toxoplasmosis, and cutaneous leishmaniasis^15,18–20^. Liang and colleagues have reported that *L. mexicana*-infected PD-L1^−/−^ mice have increased production of IFN-γ in T cells, reduced disease progression, and greater control of the parasite load when compared with infected wild-type (WT) mice. In PD-L2^−/−^ mice, however, there was an increased lesion size and increased parasite load compared to WT mice, which implies there are differing roles for PD-L1 and PD-L2 in regulating IFN-γ production. These results also suggest the participation only of PD-L1 in the T exhaustion process during *L. mexicana* infection^15^.

Recently it was demonstrated, a patient with diffuse cutaneous leishmaniasis, the expression of PD-1^+^ on CD4^+^ T cells and CD8^+^ T cell^21^. Thus, we hypothesize that the use of anti-PD-1 and anti-PD-L1 MoAbs would have the potential to reverse the T cell suppression phenotype observed in BALB/c mice. Therefore, here we investigate the expression of PD-1 and PD-L1 upon *L. amazonensis* infection in BALB/c mice, and evaluate the use of MoAbs against PD-1 and PD-L1 as therapies for the severe form of leishmaniasis caused by *L. amazonensis*.

## Materials and Methods

### Experimental animals

Female BALB/c mice, 6–8 weeks old, from the Núcleo de Animais de Laboratório (Universidade Federal Fluminense, Rio de Janeiro, Brazil), were housed in Ventilife mini-isolators (Alesco, Brazil) and kept under controlled temperature and light conditions. All of the animal experiments were performed in strict accordance with the Brazilian animal protection law (Lei Arouca number 11.794/08) of the National Council for the Control of Animal Experimentation (CONCEA, Brazil). The protocol was approved by the Committee for Animal Use of the Universidade Federal do Rio de Janeiro (Permit Number: 161/18).

### Culture of parasites

Infective promastigotes of *L. amazonensis* (MHOM/BR/75/Josefa) were obtained from infected BALB/c mouse lesions and were used until the 5^th^ culture passage as promastigotes at 26 °C in M-199 medium (Cultilab) supplemented with 20% heat-inactivated fetal bovine serum (FBS) (Cultilab).

### *In vivo* infection and treatment

BALB/c mice were infected subcutaneously in the right hind footpad with 2 × 10^6^ stationary-phase promastigotes of *L. amazonensis* in 20 µl PBS. The following antibodies were administered intraperitoneally at 100 µg in 100 µl PBS; anti-PD-L1 (BMS-936559, Bristol-Myers Squibb), anti-PD-L2 (B7-DC, clone TY25, catalog # BE0112, Bioxcell), and anti-PD-1 (CD279, clone RMP1–14, catalog # BE0146, Bioxcell). The first injection was given at 7 days post-infection. Two treatment protocols were assessed: (i) inoculation once a week for 49 days with a total of 6 doses; and (ii) twice a week for 56 days with a total of 12 doses. Control animals received 100 μl PBS intraperitoneally also at 7 days post-infection and in accordance with the two treatment protocols. For both treatments, the last dose was administered 5 days prior to the euthanasia of the animals. Footpad thickness was measured weekly by using a direct-reading Vernier caliper.

### Parasite load quantification

Parasite load was evaluated by limiting dilution assay as previously described^22^. Briefly, after the mice were euthanized, the infected paws were removed, weighed, individually macerated with a tissue mixer, the homogenate (1 ml/footpad) and submitted to serial dilution (diluted 1:4) into 96-well culture plates (final volume of 200 µl/well) and incubated at 26 °C for 15 days. The presence of promastigote cultures were examined via optical microscope (Olympus, Japan), and the last well containing promastigotes in the limiting dilution assay was recorded to calculate the parasite load.

### Cell staining for flow cytometry

Lymph nodes were individually removed and macerated with a tissue mixer.

The cytokine-production of lymph node cells was evaluated after a polyclonal activation because the LaAg induce apoptosis of T cells^23^. Cells (1 × 10^6^/well in a 24-well plate) were stimulated for 4 h at 37 °C with PMA (phorbol 12- myristate 13-acetate, 10 ng/ml, Sigma-Aldrich) and Ionomycin (10 ng/ml, Sigma-Aldrich), in the presence of a Golgi complex inhibitor Brefeldin A (5 mg/ml, Biolegend). All centrifugation steps were performed at 4 °C. Cells were washed with PBS and blocked with 50 µl/well Human FcX (BioLegend) for 15 min, after which, 50 µl/well of the staining antibody pool was added and incubated for 30 min at 4 °C. Cells were washed with buffer solution (PBS with 5% FBS) at 400 g for 5 min, then fixed and permeabilized (FoxP3 permeabilization/fixation kit; eBiosicence) according to the manufacturer’s protocol. Cells were washed again with buffer solution and resuspended in the same solution. For intracellular staining, the following antibodies were added (all used at 0.1 μg/ml) and incubated for 1 h at 4 °C in the dark: CD3 (anti-CD3-APC-780; clone 145–2C11, Biolegend), CD4 (anti-CD4-PE-Cy7; clone RM4–5, Biolegend), CD8 (anti-CD8-PerCP; clone 53-6.7, Biolegend), PD-1 (anti-PD-1-FITC; clone J43, eBiosciences), CD25 (anti CD25-PE; clone P4A10, eBioscience), CD11c (anti CD11c-PerCP; Biolegend), and intracellular IFN-γ (anti-IFN-γ-APC; clone XMG1.2, eBiosciences) and FoxP3 (clone FJK-16s; eBioscience). Cells were washed and resuspended in 150 μl buffer solution, and stored in the dark at 4 °C until acquisition. Cells were analyzed on a BD FACS CANTO II flow cytometer; data from 100,000 events were captured from cells acquired on CD3^+^ and analyzed with FlowJo^®^ software (BD-Becton, Dickinson & Company).

### Analysis of cytokines

Infected footpads were removed, and individually homogenized in 1 ml of medium using a glass tissue homogenizer, for quantification of *in situ* production. The homogenates were centrifuged (10 min, 2000 g at 4 °C), the supernatants collected and cytokines quantified by individually assaying for the presence of IL-4, IL-10, and TGF-β by specific ELISAs using a standard protocol (BD OptEIA), detection limits for IL-4 is 7.8–500 pg/ml, IL-10 is 31.3–2000 pg/ml e TGF- β is 62.5–4,000 pg/mL). The results are presented as pg/g (pg of cytokine determined by ELISA of the homogenate divided by the weight in g of the infected tissue homogenized).

### Dosage of immunoglobulins

Soluble *L. amazonensis* antigen (LaAg) was obtained from stationary phase promastigotes, which were washed 3 times in PBS, freeze-thawed for 3 cycles, lyophilized, stored at −20 °C and reconstituted with PBS just prior to use. The 96-well plates were coated with LaAg (1 μg/well) overnight at 4 °C, blocked with PBS/5% milk/0.05% Tween 20 (Sigma-Aldrich) for 2 h, and washed 3 times with PBS/0.05% Tween 20. The mouse serum samples (1:250 diluted in PBS/5% milk/0.05% Tween 20) were added. The plates were incubated at room temperature for 1 h and washed with PBS/0.05% Tween 20. Anti-IgM or anti-IgG-HRP (Southern Biotech) were added (1:2000) for 1 h at room temperature. After washing, the color was developed with a TMB solution (Life Technologies) and stopped with 1 M HCl.

### Data analysis

Results are expressed as mean ± SEM with confidence level *p* ≤ 0.05. For lesion development analysis, a two-way ANOVA with a Bonferroni post-test was used. For multiple comparisons, a one-way ANOVA followed by Tukey pairing was performed. Paired t-test analysis was done as indicated in the figure legends. Data analysis was performed using GraphPad Prism^®^ 5.00 software.

## Results

### *L. amazonensis* infection induces PD-1 expression on CD4^+^ and CD8^+^ T cells

**Figure 1:**
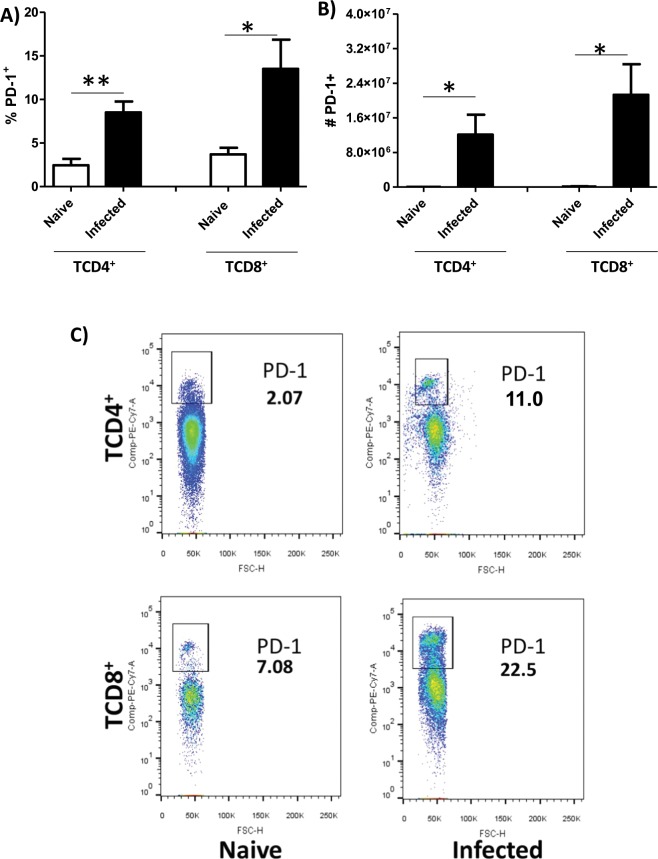
Expression of PD-1 on CD4^+^ and CD8^+^ T cells in *L. amazonensis*-infected BALB/c mice. Lymphocytes from the macerated draining popliteal lymph node of an *L. amazonensis*-infected paw were collected at 56 days post-infection. Uninfected mice were used as control. (**A,B**) Percentage and absolute number of PD-1^+^CD4^+^ T cells and PD-1^+^CD8^+^ T cells. (**C**) Dot plots showing PD-1 expression (PE-Cy7-PD-1^+^, FSC-cell volume). *p < 0.05, **p < 0.0375 (T Test). (SEM; *n* = 8–13).

**Figure 2:**
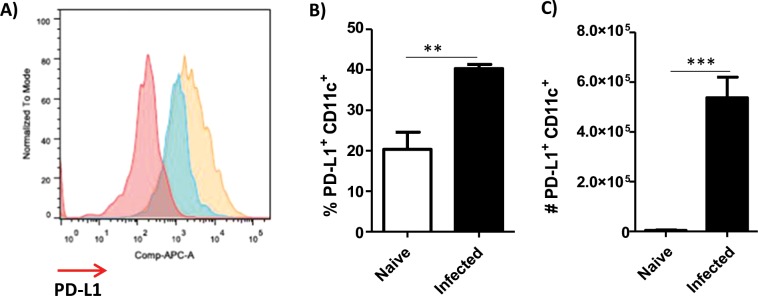
Presence of dendritic cells expressing PD-L1^+^ in the draining lymph nodes of infected mice. Mice were infected in the footpad with *L. amazonensis* promastigotes (2 × 10^6^). After approximately 2 months, the draining lymph nodes were collected, macerated and the cells were analyzed by flow cytometer. (**A**) Histogram of PD-L1 expression. Red = negative of PD-L1, Blue = naïve and Orange = PD-L1^+^ (APC-PD-L1). (**B**) Percentage of PD-L1^+^ CD11c^+^ cells. (**C**) Number of PD-L1^+^ CD11c^+^ cells. Naive = mice without infection. (T Test (**C**)). Data ± SEM from 5 mice per group are representative of two independent experiments with similar results. ***p < 0.0001.

To study the induction of PD-1 by *Leishmania amazonensis* infection in mice as observed in humans^21^, we decided to evaluate the expression of PD-1 in lymph node cells of BALB/c infected mice. Our data showed an increase in the percentage (Fig. 1(A,C)) and in the absolute numbers (Fig. 1(B)) of PD-1^+^ CD4^+^ and CD8^+^ T cells in infected compared to uninfected BALB/c mice. We also evaluated the expression of PD-L1 on dendritic cells. We observed an increase in the percentage (Fig. 2(A,B)) and numbers (Fig. 2(C)) of PD-L1 + CD11c + cells in the infected compared to uninfected BALB/c mice. These results indicated that infected mice display similar profile observed in cutaneous diffuse leishmaniasis and allowed us to study these molecules using the BALB/c mice model.

### Anti-PD-1 or anti-PD-L1 MoAb treatment reduces parasite loads without affecting lesion growth in mice

As we observed that PD-1 and PD-L1 was upregulated during *L. amazonensis* infection, we next assessed the effect of treatment with the anti-PD-1 and anti-PD-L1 blocking antibodies on the disease profile. First, *L. amazonensis-*infected BALB/c mice were treated with the individual MoAbs (100 μg each/mice) once weekly, beginning at 7 days post-infection, receiving a total of 6 doses. The footpad thickness was measured weekly, and the parasite load was determined after 49 days of treatment. We found that this therapy was not effective in modifying the lesion development profile (Supplemental Fig. 1(A) or the parasite load (Supplemental Fig. 1(B)) compared to control mice injected with PBS.

**Figure 3:**
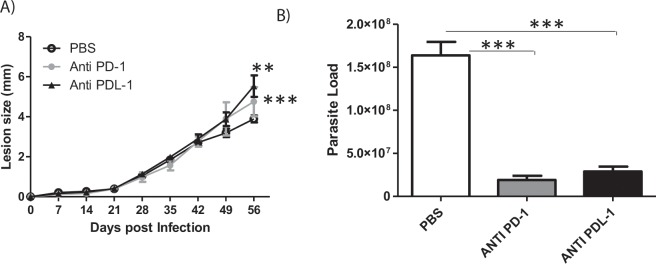
Lesion development and parasite load in *L. amazonensis*-infected BALB/c mice. Mice were infected *with L. amazonensis* promastigotes (2 × 10^6^) and treated with either anti-PD-1 or anti-PD-L1 MoAbs (100 μg/dose), administered twice a week intraperitoneally, beginning at 7 days post-infection, for 56 days. (**A**) Lesion size and (**B**) parasite load from individually mice (5 mice/group) are presented as data ± SEM of a representative experiment out of two independent one producing the same profile. **p < 0.01, ***p < 0.001(Anova (**A**)). ***p < 0.0001 (T Test (**B**)).

In the second treatment protocol, mice were given individual MoAbs twice weekly, beginning at 7 days post-infection and receiving a total of 12 doses during the 56 day observation period. We found that although the lesion sizes (Fig. 3(A)) were increased during anti-PD1 and anti-PD-L1 treatment, the parasite loads were significantly decreased after anti-PD-1 or anti-PD-L1 treatment (Fig. 3(B), Supplemental Fig. 2(B), Supplemental Fig. 3). Anti-PD-L2 treatment did not show any significant effect independent of protocol used (Supplemental Figs. 1 and 2). These results suggest the possibility of using the anti-PD-1 and anti-PD-L1 MoAb therapies to decrease parasitic load.

### Induction of IFN-γ from CD4^+^ and CD8^+^ T cells after MoAb treatment

**Figure 4:**
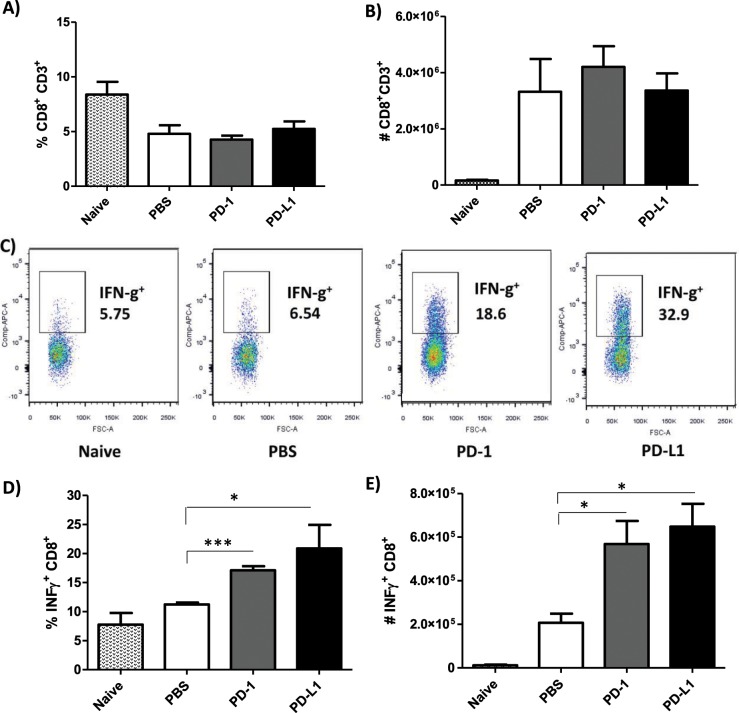
Effect of anti-PD-1 and anti-PD-L1 MoAbs on the percentage and number of IFN-γ^+^CD8^+^ T cells. Lymphocytes were collected from the draining lymph node of an *L. amazonensis*-infected paw after 56 days of treatment with anti-PD-1 or anti-PD-L1, administered twice a week intraperitoneally beginning 7 days after infection. (**A**) Percentage of CD3^+^CD8^+^ T cells. (**B**) Number of CD3^+^CD8^+^ T cells. (**C**) Dot plots showing IFN-γ expression (APC-IFN-γ, FSC-cell volume). (**D**) Percentage of IFN-γ^+^ CD8^+^ T cells. (**E**) Number of IFN-γ^+^CD8^+^ T cells. (T Test (**D**), ANOVA (**E**)). Naive = mice without infection and therapy, PBS = infected mice injected with PBS on treatment days, PD-1 = mice infected and treated with anti-PD-1 (100 μg/dose), PD-L1 = mice infected and treated with anti-PD-L1 (100 μg/dose). Data ± SEM of individually mice (5 mice/group) are representative of two independent experiments producing the same result profile. *p < 0.05, ***p < 0.0001.

Focusing on the twice a week treatment protocol, we then examined the mechanism underlying parasite load reduction in anti-PD-1 or anti-PD-L1-treated mice by measuring the IFN-γ production of CD8^+^ and CD4^+^ T cells from the draining lymph nodes. It is documented in murine models of *L. major* infection that the expression of cytokines such as IL-12 and IFN-γ by Th1 contributes to host protection, whereas IL-4, IL-5, and IL-13 expression by Th2 contributes to host susceptibility^24,25^. In the murine *L. amazonensis* infection model, we and others have shown that impaired IFN-γ production and insufficient macrophage activation favor parasite survival and persistence^24–27^. In our treatment studies, we found more CD3^+^CD8^+^ T cells in the *L. amazonensis-*infected mice compared to uninfected mice (Fig. 1(A)), but there were no significant difference in the percentage and absolute number of CD3^+^CD8^+^ T cells between the MoAb-treated and the PBS-injected groups (Fig. 4(A,B)). However, both, percentage and absolute number of IFN-γ-producing CD3^+^CD8^+^ T cells (IFN-γ^+^CD8^+^ T cells) were significantly increased in anti-PD-1 or anti-PD-L1-treated mice when compared to the PBS-injected group (Fig. 4(C–E).

**Figure 5:**
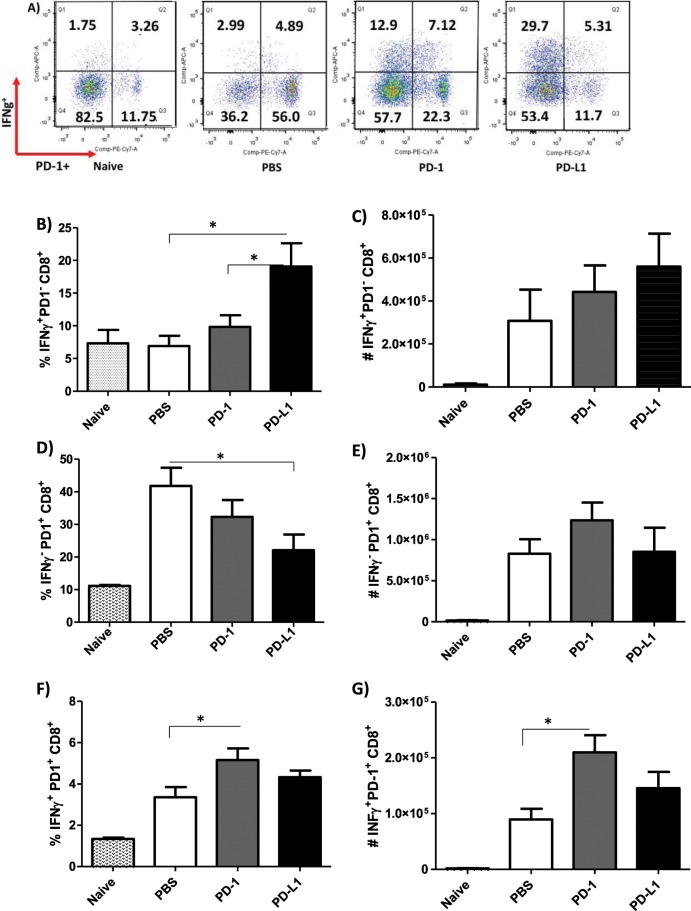
Increase of IFN-γ^+^CD8^+^ T cells after anti-PD-1 and anti-PD-L1 MoAb treatment. Lymphocytes were collected from the popliteal lymph node of an *L. amazonensis*-infected paw after approximately 2 months of treatment with anti-PD-1 or anti-PD-L1, administered twice a week intraperitoneally beginning 7 days after infection. (**A**) Dot plot IFN-γ and PD-1 expression (APC-IFN-γ, PE-Cy7-PD-1). (**B**) Percentage of IFN-γ^+^PD-1^−^CD8^+^ T cells. (**C**) Number of IFN-γ^+^PD-1^−^ CD8^+^T cells. (**D**) Percentage of IFN-γ^−^PD-1^+^CD8^+^T cells. (**E**) Number of IFN-γ^−^PD-1^+^ CD8^+^T cells. (**F**) Percentage of IFN-γ^+^PD-1^+^CD8^+^ T cells. (**G**) Number of IFN-γ^+^PD-1^+^CD8^+^ T cells. (T Test). Naive = mice without infection and therapy, PBS = infected mice injected with PBS on treatment days, PD-1 = mice infected and treated with anti-PD-1 (100 μg/dose), PD-L1 = mice infected and treated with anti-PD-L1 (100 μg/dose). Data ± SEM of individually mice (5 mice/group) are representative of two independent experiments producing the same result profile. *p < 0.05.

As the percentage and number of PD-1^+^CD8^+^ T cells were increased in *L. amazonensis* infection (Fig. 1), we next examined whether the IFN-γ production could also be affected in the CD8^+^ T cells lacking PD-1 (PD-1^−^CD8^+^) during MoAb treatment. Similar to the total CD3^+^CD8^+^ T cells, the PD-1^−^CD8^+^ T cells of both groups of MoAb-treated mice presented a higher percentage of IFN-γ expression, but there was not a significantly higher number of IFN-γ^+^PD-1^−^CD8^+^ T cells compared to the PBS-injected group (Fig. 5(A–C)).

Regarding PD-1^+^CD8^+^ T cells not expressing IFN-γ (IFN-γ ^−^PD-1^+^CD8^+^), there was a significant decrease in the percentage of these cells in anti-PD-L1-treated mice compared to the PBS-injected group, without any effect on the absolute numbers (Fig. 5(D,E)). We did observe an increase in the IFN-γ-expressing PD-1^+^CD8^+^ T cells in both the percentage and absolute number of anti-PD-1-treated mice, but there was no significant difference in anti-PD-L1-treated mice compared to the PBS-injected group (Fig. 5(F,G)).

**Figure 6:**
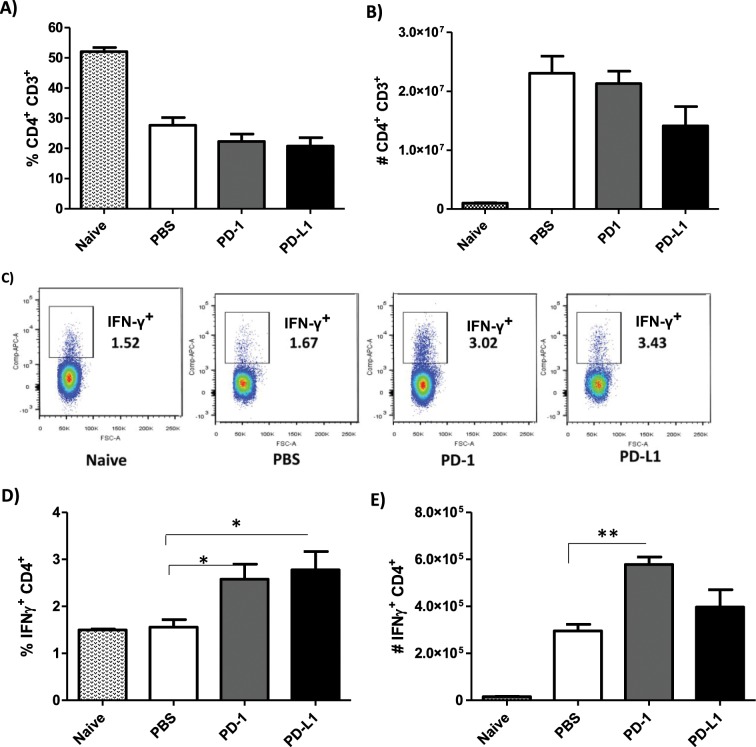
Increase of IFN-γ^+^CD4^+^ T cells after anti-PD-1 and anti-PD-L1 MoAb treatment. Lymphocytes were collected from the popliteal lymph node of an *L. amazonensis*-infected paw after approximately 2 months of treatment with anti-PD-1 or anti-PD-L1, administered twice a week intraperitoneally beginning after 7 days of infection. (**A**) Percentage of CD3^+^CD4^+^ T cells. (**B**) Number of CD3^+^CD4^+^ T cells. (**C**) Dot plot of IFN-γ expression (APC-IFN-γ, FSC-cell volume). (**D**) Percentage of IFN-γ^+^CD4^+^ T cells. (**E**) Number of IFN-γ^+^ CD4^+^ T cells. (T Test (**D**) and ANOVA (**E**)). Naive = mice without infection and therapy, PBS = infected mice injected with PBS on treatment days, PD-1 = mice infected and treated with anti-PD-1 (100 μg/dose), PD-L1 = mice infected and treated with anti-PD-L1 (100 μg/dose). Data ± SEM of individually mice (5 mice/group) are representative of two independent experiments producing the same result profile. *p < 0.05, **p < 0.0375.

We also investigated the profile of CD4^+^ T cells and found no major differences between the MoAb-treated and PBS-injected groups regarding the percentage and number of CD3^+^CD4^+^ T cells (Fig. 6(A,B)). Like the CD3^+^CD8^+^ T cells, the percentage of CD4^+^ T cells producing IFN-γ was significantly increased after treatment with anti-PD-1 and anti-PD-L1 (Fig. 6(C,D)), however, a significant increase in the number of IFN-γ^+^-producing CD4^+^ T cells was observed in the anti-PD-1 treatment (Fig. 6(E)).

**Figure 7:**
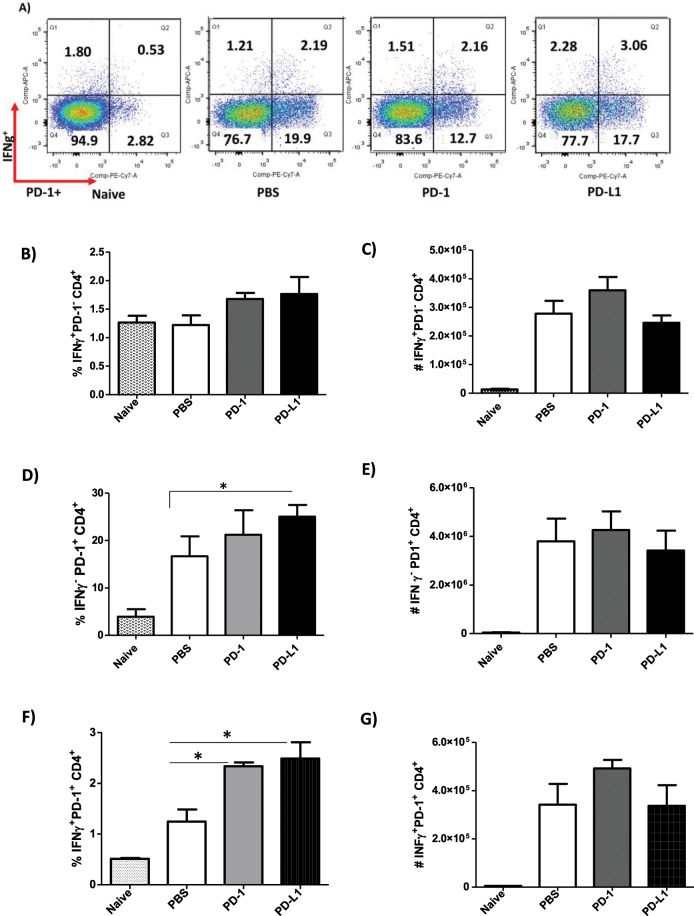
Increase in the percentage of IFN-γ^+ ^PD-1^+^CD4^+^ T cells after anti-PD-1 and anti-PD-L1 MoAb treatment. Lymphocytes were collected from the popliteal lymph node of an *L. amazonensis*-infected paw after approximately 2 months of treatment with anti-PD-1 or anti-PD-L1, administered twice a week intraperitoneally beginning after 7 days of infection. (**A**) Dot plot of IFN-γ and PD-1 expression (APC-IFN-γ, PE-Cy7-PD-1). (**B**) Percentage of IFN-γ^+^PD-1^−^ CD4^+^ T cells. (**C**) Number of IFN-γ^+ ^PD-1^− ^CD4^+^ T cells. (**D**) Percentage of IFN-γ^− ^PD-1^+^ CD4^+^ T cells. (**E**) Number of IFN-γ^− ^PD-1^+^ CD4^+^ T cells. (**F**) Percentage of IFN-γ^+ ^PD-1^+^ CD4^+^ T cells. (**G**) Number of IFN-γ^+ ^PD-1^+^ CD4^+^ T cells. (T Test (**D**) and ANOVA (**F**)). Naive = mice without infection and therapy, PBS = infected mice injected with PBS on treatment days, PD-1 = mice infected and treated with anti-PD-1 (100 μg/dose), PD-L1 = mice infected and treated with anti-PD-L1 (100 μg/dose). Data ± SEM of individually mice (5 mice/group) are representative of two independent experiments producing the same result profile. *p < 0.05.

Unlike the PD-1^−^CD8^+^ T cells, MoAb treatment had no effect on the IFN-γ production by PD-1^−^CD4^+^ T cells (Fig. 7(A–C)). Again, opposite to what was observed in the IFN-γ^−^PD-1^+^CD8^+^ T cells, there was a significantly higher percentage of IFN-γ^− ^PD-1^+^CD4^+^ T cells in anti-PD-L1-treated mice, with no difference on the absolute number of these cells (Fig. 7(D,E)). Additionally, a significant increase in the percentage of IFN-γ-producing PD-1^+^CD4^+^ T cells (IFN-γ^+ ^PD-1^+^CD4^+^) was found for both MoAb-treated groups compared to the PBS-infected group, which was not observed in the number of cells (Fig. 7(F,G)). We did not observe any difference in the production of IFN-γ^+^ or expression of PD-1^+^ by CD3^+^CD4^−^CD8^−^ (Supplemental Figs. 6 and 7).

Altogether, our results suggest that anti-PD-1 and anti-PD-L1 MoAb treatment stimulated the production of IFN-γ in both CD4^+^ and CD8^+^ T cells, which may be one of the possible mechanisms for the control of parasite load.

### Anti-PD-1 treatment reduces IL-4 and TGF-β

**Figure 8:**
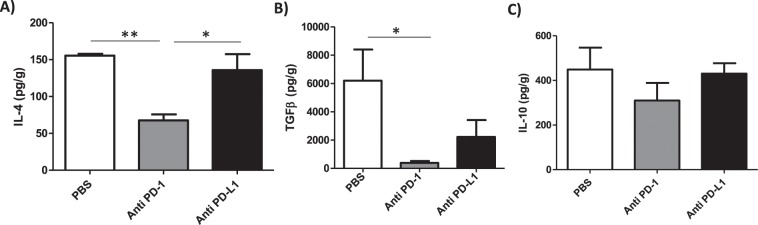
Selective reduction of IL-4 and TGF-β in MoAb-treated groups. *L. amazonensis*-infected paws were collected and macerated after approximately 2 months of treatment with anti-PD-1 or anti-PD-L1, administered intraperitoneally twice a week beginning after 7 days of infection, and the supernatant was analyzed by ELISA: (**A**) IL-4, (**B**) TGF-β, (**C**) IL-10. Data ± SEM of individually mice (5 mice/group) are representative of two independent experiments producing the same result profile. *p < 0.05, **p < 0.0375 (ANOVA).

As IFN-γ production was induced by the MoAb treatment, we tested the effect of treatment on the modulation of the cytokines, IL-4, IL-10 and TGF-β, at the site of *L. amazonensis* infection. Only anti-PD-1 treatment significantly decreased IL-4 (Fig. 8(A)) and TGF-β (Fig. 8(B)) production *in situ* compared to the PBS-injected control group. No alteration in the IL-10 levels was found after MoAb treatment (Fig. 8(C)). Finally, we demonstrated that treatment with the MoAbs did not affect the production of anti-*Leishmania* specific IgM or IgG antibodies as assessed in the blood sera (Supplemental Fig. 4(A,B)). Altogether, our results suggest that anti-PD-1 MoAb treatment modulates the production of IFN-γ in CD4^+^ T and CD8^+^ T cells, but anti-PD-L1 only affects CD8^+^ T cells.

## Discussion

The use of leukocyte receptor blockers in immunotherapy has been extensively studied in oncology^28^. One of the clinical trials concerning the systemic administration of therapeutic antibodies to block PD-1 or PD-L1 has produced promising results for the treatment of several tumors^29,30^. However, the use of this therapy is still relatively limited in non-healing leishmaniasis.

For *L. major* infection in arginase-deficient mice, deficiency in T cell activation resulted in increased PD-1 expression, impairing the immune response and inducing T cell exhaustion^31^. In dogs injected with *L. infantum* antigens, PD-L1/PD-1 blockade with specific antibodies recovered the proliferation of CD4^+^ and CD8^+^ T cells, in addition to the production of IFN-γ by CD4^+^ T cells^32^. In studies of *L. donovani* infection in BALB/c mice, the parasite induced the initial expansion of IFN-γ-producing CD4^+^ and CD8^+^ T cells in the acute phase of the disease, the frequency of which was reduced after 21 days post-infection even with a robust parasite presence. In this model, blocking PD-L1 resulted in the restoration of CD4^+^ and CD8^+^ T cell responses, leading to a reduction in parasite load^33^. In view of these studies, our results presented herein show that *L. amazonensis* infection interferes in the production of IFN-γ by both CD4^+^ and CD8^+^ T cells.

Recently, the presence of PD-1 and PD-L1 was detected in a patient with diffuse cutaneous leishmaniasis caused by *L. amazonensis*^21^. Based on the induction of PD-1 and PD-L1 by *Leishmania* infection, the blocking of these molecules may be a new strategy to treat leishmaniasis.

The use of anti-PD-1 and anti-PD-L1 antibodies in clinical cancer treatment studies, such as in pancreatic tumor, were administered at doses between 10 and 200 μg every three days^34–36^. In our study, we tested a lower therapeutic dose of 100 µg of the anti-PD-L1, anti-PD-L2 and anti-PD-1 antibodies once a week. However, our data revealed that this was insufficient in reducing the lesion size or parasite load. Therefore, the dosage of the treatment, in terms of the concentration and frequency of administration are important considerations. Thus, we increased the administration to twice a week, and observed that although the lesion size was increased, the parasite load in the infected footpads, in the spleen and in the draining lymph nodes that received therapy with anti-PD-1 and anti-PD-L1 were controlled.

In *L. major* infection, only treatment with 1 mg/dose of the anti-PD-1 antibody weekly in infected arginase-deficient mice led to complete resolution of the chronic skin lesion and resistance to infection^31^. A similar result was found when using the anti-PD-L1 antibody in mice challenged with *L. donovani* amastigotes, as these mice showed a reduction of up to 87% of the parasite load in the spleen^37^. The susceptibility of the C57BL/10 and C57BL/6 mice to infection by *L. amazonensis* is related to the absence of Th1 type cellular immune response and not controlled exclusively by Th2 cells^38,39^. In BALB/c mice, this susceptibility is related to Th2 type cellular immune response^40,41^.

In this study, we observed that the production of IFN-γ was higher in CD8^+^ T lymphocytes, both in percentage and number of cells after treatment with the anti-PD-1 and anti-PD-L1 MoAbs. We also detected an increase in the percentage of IFN-γ-producing CD4^+^ T lymphocytes after both treatments. However, when looking at the number of IFN-γ-producing CD4^+^ T cells only in mice treated with anti-PD-1 a significant increase was observed. These results suggest that treatment with anti-PD-1, acting directly on the lymphocytes, is more competent in invigorating CD4^+^ T lymphocytes than the anti-PD-L1 therapy, the target of which is in the antigen-presenting cells. Our data indicate that the increase of IFN-γ is one of the possible mechanisms of the therapeutic efficacy of these monoclonal antibody therapies, mainly by CD8^+^ T and partially by CD4^+^ T lymphocytes.

The PD-1/PD-L1 ratio is very important in suppressing the CD8^+^ T cell response during *L. donovani* infection^37^. In a study of dogs with symptomatic visceral leishmaniasis by *L. infantum*, these animals were shown to have a five-fold reduction in the proliferative capacity of CD8^+^ T cells and a reduction of up to three-fold in the ability of these cells to produce IFN-γ. After administration of specific monoclonal antibody therapy, PD-1 blockade significantly increased the proliferative capacity of CD4^+^ and CD8^+^ T cell populations, and recovered IFN-γ production in the CD4^+^ population. In addition, T cell depletion during visceral leishmaniasis was associated with elevated expression of PD-1, which could be identified before the onset of the disease and is considered a determining factor for symptomatic onset^32^. In another study of canine visceral leishmaniasis, it was observed that as the disease progressed, there was a decrease in CD4^+^ T cell proliferation and also a reduction of IFN-γ production in response to *L. infantum* antigens^40^.

These findings are reinforced by studies in which anti-PD-1 and anti-PD-L1 therapy reverts the ability of CD8^+^ T lymphocytes to produce IFN-γ as in the treatment of thyroid cancer^41^, in chronic hepatitis B^42^, and in HIV infection^43^. Further studies should be performed to confirm whether T cell exhaustion occurs in *L. amazonensis* infection. However, other studies that have assessed how chronic infections can induce exhaustion, in addition to our results reporting the increased capacity of IFN-γ production after MoAbs therapy, this suggests that T cell exhaustion occurs in *L. amazonensis* infection.

The participation of IFN-γ has been reported as playing a dual effect in *L. amazonensis* infection when discussing inflammatory response/lesion versus parasite load. IFN-γ participates in the control of parasite load enhancing the killing; however, it is also related to cell recruitment that increases the inflammatory response contributing to lesion development in WT mice^44^. In our experiments, in BALB/c mice, we demonstrated a similar mechanism, together with the increase of IFN-γ, we observed a decreased of parasite load, however, a small increase of the lesion associated to increase of inflammatory response that is also accompanying by the parasite load control.

Interestingly, our results indicate the potential of MoAb to modulate the cytokines present at the lesion site in the mice, as the production of IL-4 and TGF-β were reduced in the group treated with the anti-PD-1 MoAb. The group treated with anti-PD-L1 MoAb showed a tendency in the reduction of TGF-β.

It is already known that patients who progress to the disease have an increase in the production of immunosuppressive responses by the increase of TGF-β in *Leishmania* infection^45,46^. TGF-β also plays a role in susceptibility of murine cutaneous leishmaniasis caused by *L. amazonensis*^47^ and *L. chagasi* infection^48^. TGF-β was shown to upregulate the PD-L1 expression in dendritic cells, leading to T-cell anergy and diminished anti-tumor response^49^. Thus, our data support the hypothesis that IFN-γ produced by CD4^+^ T cells reduces TGF-β production. Moreover, it is important to point out that TGF-β is associated with increased parasite load^45^ and its reduction is directly related to parasite control. Hence, in our results, parasitic control may also be related to the decrease of TGF-β.

We also evaluated the production of IL-10 and IL-4 in this model. IL-10 has been reported to be associated to pathogenesis in *L. mexicana*, *L. amazonensis*^50^, *L. donovani*^51^ and *L infantum*^40^ infections. The participation of IL-10 was not seen in our results, since the concentration of IL-10 showed only a slight reduction in the MoAb-treated groups relative to the PBS-injected control, thus the reduction in parasite load is likely not associated to the levels of IL-10 in our model. We observed the reduction of IL-4 in anti-PD-1 treated mice. It is possible that the IFN-γ produced by CD4^+^ T cells may inhibit the development of a Th2 response reducing IL-4 production. IL-4 has been associated linked to lesion pathogenesis in murine cutaneous leishmaniasis caused by *L. amazonensis*, but does not directly affect the parasite load^52^. In human diffuse cutaneous leishmaniasis, there is no persistence of Th2 response in the lesions^53^. These data suggested that the reduction of IL-4 perhaps is not related to control of parasite load in our model.

We also evaluated if the humoral response was altered during MoAb treatment. Kima et al. showed that antibodies play a critical role in the pathogenesis and in the development of more significant lesions due to *L. amazonensis* infection, since the maintenance of infection by these parasites was impaired by the absence of circulating antibodies in the BALB/c model^54^. Recently, we demonstrated that *L. amazonensis* infection in XID mice displayed smaller lesions and a decrease in IL-10 and total antibodies in comparison to WT mice suggesting the pathogenic role of B cells in *L. amazonensis* infection^55^. Here, we demonstrated that treatment with both MoAbs did not affect the anti-*Leishmania* IgM, IgG, IgG1 and IgG2a antibodies levels.

In summary, our study suggests a potential use of monoclonal antibodies against PD-1 and PD-L1 in the treatment of cutaneous leishmaniasis caused by *L. amazonensis*. Our model, which employed a low dose treatment of anti-PD-1 or anti-PD-L1 showed therapeutic efficacy to control the parasite load in infected mice. We have also shown that this control is related to CD8^+^ T lymphocytes and, partially, to CD4^+^ T lymphocytes, producing IFN-γ. These findings could potentiate a combined therapy using anti-PD-1 or anti-PD-L1 antibodies and the current standard therapies against leishmaniasis, which could be particularly important for diffuse cutaneous leishmaniasis treatment, a disease that is refractory to conventional treatment.

## Conclusion

The present work analyzed the use of monoclonal antibodies as a potential treatment of cutaneous leishmaniasis caused by *L. amazonensis*. Our *L. amazonensis* mouse infection model presents a susceptible immune response and our treatment protocol with the MoAbs against PD-1 and PD-L1 favored the control of parasitic burden. These data together indicate that the monoclonal antibody therapy targeting PD-1 or PD-L1 could be used as a possible treatment for leishmaniasis..

## Supplementary information

Supplementary Figures

## References

[1] Ishida Y, Agata Y, Shibahara K, Honjo T (1992). Induced expression of PD-1, a novel member of the immunoglobulin gene superfamily, upon programmed cell death. The EMBO journal.

[2] Torres-Guerrero E, Quintanilla-Cedillo MR, Ruiz-Esmenjaud J, Arenas R (2017). Leishmaniasis: a review. F1000Research.

[3] Dong H (1999). B7-H1, a third member of the B7 family, co-stimulates T-cell proliferation and interleukin-10 secretion. Nat Med.

[4] Nishimura H, Honjo T (2001). PD-1: An inhibitory immunoreceptor involved in peripheral tolerance. Trends Immunol..

[5] Wang W (2009). PD1 blockade reverses the suppression of melanoma antigen-specific CTL by CD4 + CD25(Hi) regulatory T cells. International immunology.

[6] Guerin LR, Prins JR, Robertson SA (2009). Regulatory T-cells and immune tolerance in pregnancy: a new target for infertility treatment?. Human reproduction update.

[7] Iwai Y (2002). Involvement of PD-L1 on tumor cells in the escape from host immune system and tumor immunotherapy by PD-L1 blockade. Proceedings of the National Academy of Sciences of the United States of America.

[8] Zou W, Chen L (2008). Inhibitory B7-family molecules in the tumour microenvironment. Nat Rev Immunol.

[9] Curiel T (2003). Blockade of B7-H1 improves myeloid dendritic cell–mediated antitumor immunity. Nat Med.

[10] Hino R (2010). Tumor cell expression of programmed cell death‐1 ligand 1 is a prognostic factor for malignant melanoma. Cancer.

[11] Taube JM (2012). Colocalization of inflammatory response with B7-h1 expression in human melanocytic lesions supports an adaptive resistance mechanism of immune escape. Science translational medicine.

[12] Patel, R., Bock, M., Polotti, C. F. & Elsamra, S. Pharmacokinetic drug evaluation of atezolizumab for the treatment of locally advanced or metastatic urothelial carcinoma. Expert Opin Drug Metab Toxicol. Feb;**13**(2):225–232. Epub. Jan 11. doi:10.1080/17425255.2017.1277204 (2017). 10.1080/17425255.2017.1277204 28043166

[13] Wang C, Yu X, Wang W (2016). A meta-analysis of efficacy and safety of antibodies targeting PD-1/PD-L1 in treatment of advanced nonsmall cell lung cancer. Medicine.

[14] Nikolova M (2016). Subset- and Antigen-Specific Effects of Treg on CD8^+^ T Cell Responses in Chronic HIV Infection. PLoS Pathogens.

[15] Liang SC (2006). PD-L1 and PD-L2 have distinct roles in regulating host immunity to cutaneous leishmaniasis. Eur. J. Immunol..

[16] Latchman Y (2001). PD-L2 is a second ligand for PD-1 and inhibits T cell activation. Nat Immunol.

[17] Zhang Y (2006). Regulation of T cell activation and tolerance by PDL2. Proceedings of the National Academy of Sciences of the United States of America..

[18] Bhadra R, Gigley JP, Weiss LM, Khan IA (2011). Control of Toxoplasma reactivation by rescue of dysfunctional CD8 + T-cell response via PD-1-PDL-1 blockade. Proceedings of the National Academy of Sciences of the United States of America.

[19] Wherry E (2011). T cell exhaustion. Nat Immunol.

[20] Hernández-Ruiz J (2010). CD8 cells of patients with diffuse cutaneous leishmaniasis display functional exhaustion: the latter is reversed. in vitro, by TLR2 agonists. PLoS neglected tropical diseases.

[21] Barroso DH (2018). PD-L1 May Mediate T-Cell Exhaustion in a Case of Early Diffuse Leishmaniasis Caused by *Leishmania* (L.) *amazonensis*. Frontiers in immunology.

[22] Torres-Santos EC, Rodrigues JM, Moreira DL, Kaplan MA, Rossi-Bergmann B (1999). Improvement of *in vitro* and *in vivo* antileishmanial activities of 2′, 6′-dihydroxy-4′-methoxychalcone by entrapment in poly(D,L-lactide) nanoparticles. Antimicrobial agents and chemotherapy.

[23] Pinheiro, R. O., Pinto, E. F., Benedito, A. B., Lopes, U. G. & Rossi-Bergmann, B. The T-cellanergy induced by Leishmania amazonensis antigens is related with defectiveantigen presentation and apoptosis. An Acad Bras Cienc. Sep;**76**(3):519–27.Epub. 10.1590/S0001-37652004000300006 (2004). 10.1590/s0001-37652004000300006 15334250

[24] Scott P (1989). The role of Th1 and Th2 cells in experimental cutaneous leishmaniasis. Exp. Parasitol..

[25] Heinzel FP, Sadick MD, Holaday BJ, Coffman RL, Locksley RM (1989). Reciprocal expression of interferon gamma or interleukin 4 during the resolution or progression of murine leishmaniasis. Evidence for expansion of distinct helper T cell subsets. The Journal of experimental medicine.

[26] Awasthi A, Mathur. RK, Saha B (2004). Immune response to *Leishmania* infection. Indian Journal of Medical Research..

[27] Qi H, Ji J, Wanasen N, Soong L (2004). Enhanced replication of Leishmania amazonensis amastigotes in gamma interferon-stimulated murine macrophages: implications for the pathogenesis of cutaneous leishmaniasis. Infection and immunity.

[28] Marin-Acevedo JA (2018). Next generation of immune checkpoint therapy in cancer: new developments and challenges. Journal of hematology & oncology..

[29] Topalian SL (2012). Safety, activity, and immune correlates of anti-PD-1 antibody in cancer. The New England journal of medicine.

[30] Brahmer JR (2012). Safety and activity of anti-PD-L1 antibody in patients with advanced cancer. N Engl J Med..

[31] Mou ZM (2013). Parasite-derived arginase influences secondary anti-Leishmania immunity by regulating programmed cell death-1-mediated CD4+ T cell exhaustion. Journal of immunology (Baltimore, Md.:1950).

[32] Esch KJ, Juelsgaard R, Martinez PA, Jones DE, Petersen CA (2013). Programmed death 1-mediated T cell exhaustion during visceral leishmaniasis impairs phagocyte function. Journal of immunology (Baltimore, Md.:1950).

[33] Habib S (2018). PDL-1 Blockade Prevents T Cell Exhaustion, Inhibits Autophagy, and Promotes Clearance of Leishmania donovani. Infection and immunity..

[34] Shindo, Y. *et al*. Combination immunotherapy with 4-1BB activation and PD-1 blockade enhances antitumor efficacy in a mouse model of subcutaneous tumor. *Anticancer Res*. Jan;**35**(1):129–36 2015.. 25550543

[35] Blake, S. J. P. *et al*. Blockade of PD-1/PD-L1 Promotes Adoptive T-Cell Immunotherapy in a Tolerogenic Environment. Labrecque N, ed. *PLoS One*., **10**(3):e0119483. 10.1371/journal.pone.0119483 (2015). 10.1371/journal.pone.0119483 PMC4351071 25741704

[36] Soares KC (2015). PD-1/PD-L1 blockade together with vaccine therapy facilitates effector T cell infiltration into pancreatic tumors. Journal of immunotherapy. (Hagerstown, Md:1997)..

[37] Joshi, T. *et al*. B7-H1 blockade increases survival of dysfunctional CD8(+) T cells and confers protection against Leishmania donovani infections. PLoS Pathog. May;**5**(5): e1000431. 10.1371/journal.ppat.100043 (2009). 10.1371/journal.ppat.1000431 PMC2674929 19436710

[38] Afonso LC, Scott P (1993). Immune response associated with susceptibility of C57BL/10 mice to *Leishmania amazonensis*. Infect Immun..

[39] Soong L (1997). Role of CD4^+^ T cells in pathogenesis associated with *Leishmania amazonensis* infection. J Immunol..

[40] Boggiatto PM (2010). Immunologic indicators of clinical progression during canine Leishmania infantum infection. Clinical and vaccine immunology: CVI.

[41] Bastman JJ (2016). Tumor-infiltrating T Cells and the PD-1 checkpoint pathway in advanced differentiated and anaplastic thyroid cancer. Journal of Clinical Endocrinology & Metabolism..

[42] Nebbia G (2012). Upregulation of the Tim-3/galectin-9 pathway of T cell exhaustion in chronic hepatitis B virus infection. PloS one.

[43] Trautmann L (2006). Upregulation of PD-1 expression on HIV-specific CD8^+^ T cells leads to reversible immune dysfunction. Nat Med.

[44] Carneiro MB (2015). IFN-γ-Dependent Recruitment of CD4(+) T Cells and Macrophages Contributes to Pathogenesis During Leishmania amazonensis Infection. J Interferon Cytokine Res..

[45] Barral-Netto M (1992). Transforming growth factor-β in leishmanial infection: a parasite escape mechanism. Science.

[46] Barral A (1995). Transforming growth factor-β in human cutaneous leishmaniasis. Am J Pathol. Oct.

[47] Pinheiro Roberta Olmo, Pinto Eduardo Fonseca, Lopes Janaina Ribeiro Correia, Guedes Herbert Leonel Matos, Fentanes Regina Ferro, Rossi-Bergmann Bartira (2005). TGF-β-associated enhanced susceptibility to leishmaniasis following intramuscular vaccination of mice with Leishmania amazonensis antigens. Microbes and Infection.

[48] Wilson Mary?E., Recker Thomas?J., Rodriguez Nilda?E., Young Betty?M., Burnell Kindra?K., Streit Judy?A., Kline Joel?N. (2002). The TGF-? response toLeishmania chagasi in the absence of IL-12. European Journal of Immunology.

[49] Song Shasha, Yuan Pingfan, Wu Huaxun, Chen Jingyu, Fu Jingjing, Li Peipei, Lu Jingtao, Wei Wei (2014). Dendritic cells with an increased PD-L1 by TGF-β induce T cell anergy for the cytotoxicity of hepatocellular carcinoma cells. International Immunopharmacology.

[50] Padigel, U. M., Alexander, J. & Farrell, J .P. The role of interleukin-10 in susceptibility of BALB/c mice to infection with Leishmania mexicana and Leishmania amazonensis. J Immunol. Oct **1**;171(7):3705–10 (2003). 10.4049/jimmunol.171.7.3705 14500669

[51] Nylen S (2007). Splenic accumulation of IL-10 mRNA in T cells distinct from CD4^+^CD25^+^ (Foxp3) regulatory T cells in human visceral leishmaniasis. J Exp Med..

[52] Felizardo Tania C., Gaspar-Elsas Maria I.C., Lima Gloria M.C.A., Abrahamsohn Ises A. (2012). Lack of signaling by IL-4 or by IL-4/IL-13 has more attenuating effects on Leishmania amazonensis dorsal skin – than on footpad-infected mice. Experimental Parasitology.

[53] Christensen Stephen M., Belew Ashton T., El-Sayed Najib M., Tafuri Wagner L., Silveira Fernando T., Mosser David M. (2019). Host and parasite responses in human diffuse cutaneous leishmaniasis caused by L. amazonensis. PLOS Neglected Tropical Diseases.

[54] Kima PE (2000). Internalization of *Leishmania mexicana* complex amastigotes via the Fc receptor is required to sustain infection in murine cutaneous leishmaniasis. Journal of Experimental Medicine..

[55] Firmino-Cruz Luan, Ramos Tadeu Diniz, da Fonseca-Martins Alessandra Marcia, Maciel-Oliveira Diogo, Oliveira-Silva Gabriel, Pratti Juliana Elena Silveira, Cavazzoni Cecília, Chaves Suzana Passos, Oliveira Gomes Daniel Claudio, Morrot Alexandre, Freire-de-Lima Leonardo, Vale André M., Freire-de-Lima Celio Geraldo, Decote-Ricardo Debora, de Matos Guedes Herbert Leonel (2018). Immunomodulating role of IL-10-producing B cells in Leishmania amazonensis infection. Cellular Immunology.

