# Supplementary material for: Immunotherapy using anti-PD-1 and anti-PD-L1 in Leishmania amazonensis-infected BALB/c mice reduce parasite load
Source: Sci Rep. 2019 Dec 30;9:20275. doi: 10.1038/s41598-019-56336-8 (PMC6937231; doi:10.1038/s41598-019-56336-8)

Immunotherapy using anti-PD-1 and anti-PD-L1 in *Leishmania amazonensis*-infected BALB/c mice reduce parasite load.

Alessandra M. da Fonseca-Martins, Tadeu D. Ramos, Juliana E.S. Pratti, Luan Firmino-Cruz, Daniel Claudio Oliveira Gomes, Lynn Soong, Elvira M. Saraiva, Herbert L. de Matos Guedes*

* Corresponding author: Herbert Leonel de Matos Guedes

E-mail: herbert@biof.ufrj.br, herbert@xerem.ufrj.br, herbert@ioc.fiocruz.br

Supplementary Information:

Supplementary Figure 1: Ineffectiveness of treatment with a weekly dose of specific antibodies.

Supplementary Figure 2: Unaltered lesion growth and control of parasite load from two weekly doses of specific antibodies.

Supplementary Figure 3: Control of parasite load from two weekly doses of specific antibodies.

Supplementary Figure 4: Effects of MoAb treatment on the specific anti-*Leishmania* immunoglobulins.

Supplementary Figure 5: Treg cells in *L. amazonensis*-infected BALB/c mice.

Supplementary Figure 6: Analyse of IFN-γ production on $CD3^{+}CD4^{-}$ $CD8^{-}$.

Supplementary Figure 7: Analyse of IFN-γ^{+}PD-1^{+} $CD4^{-}$ $CD8^{-}$ T cells after anti-PD-1 and anti-PD-L1 MoAb treatment.

Supplementary Figure 8: Strategy of gate of CD4 and CD8 T cells.

Supplementary Figure 9: Strategy of gate of PD-$L1^{+}$ $CD11c^{+}$.

Suppl. Fig. 1: Ineffectiveness of treatment with a weekly dose of specific antibodies. Mice were infected in the footpad with *L. amazonensis* promastigotes ($2x10^6$) and treated with anti-PD-1, anti-PD-L1 or anti-PD-L2, all at a 100 µg/dose, administered once a week intraperitoneally beginning at 7 days post-infection. (A) Progression of the lesion. (B) Parasite load. Data ± SEM of individually mice (5 mice/group) .

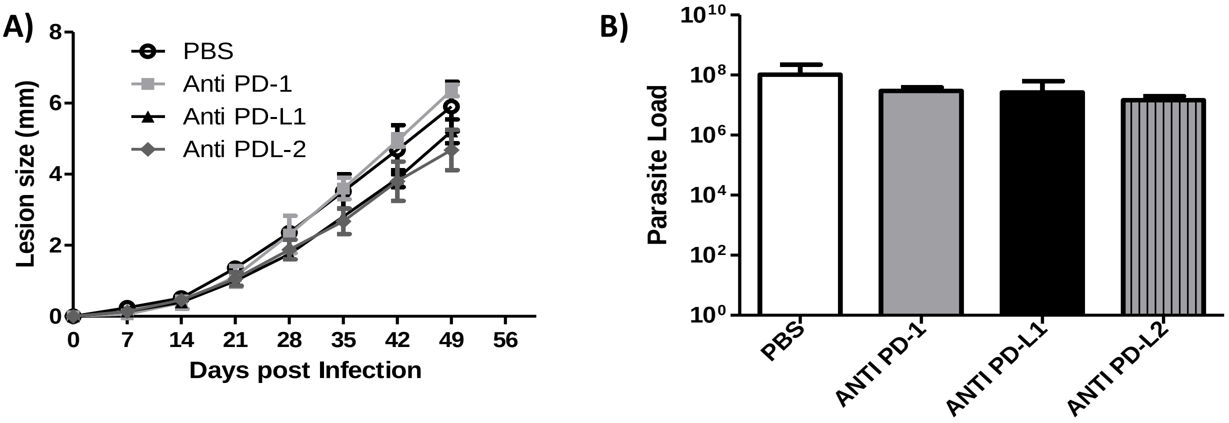

Suppl. Fig. 2: Unaltered lesion growth and control of parasite load from two weekly doses of specific antibodies. Mice were infected in the footpad with *L. amazonensis* promastigotes (2x10^6) and treated with anti-PD-1, anti-PD- L1 or anti-PD-L2, all at a 100 µg/dose, administered twice per week intraperitoneally beginning at 7 days post-infection. (A) Progression of the lesion. (B) Parasite load of paw analyzed by limiting dilution. Data ± SEM of individually mice (5 mice/group). ***p <0.0001 (T Test).

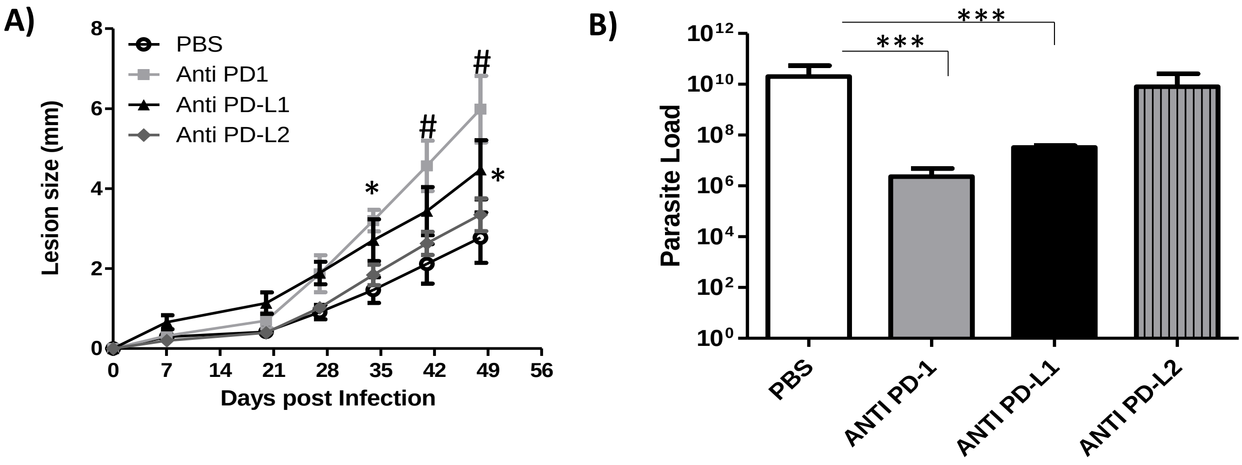

Suppl. Fig. 3: Control of parasite load from two weekly doses of specific antibodies. Mice were infected in the footpad with *L. amazonensis* promastigotes ($2x10^6$) and treated with anti-PD-1, anti-PD-L1, all at a 100 μg/dose, administered twice per week intraperitoneally beginning at 7 days post-infection. (A) Parasite load of draining lymph node. (B) Parasite load of spleen. Data ± SEM of individually mice (5 mice/group). *p<0.05, **p<0.0375. (T test (A) and Mann Whitney test. (B)).

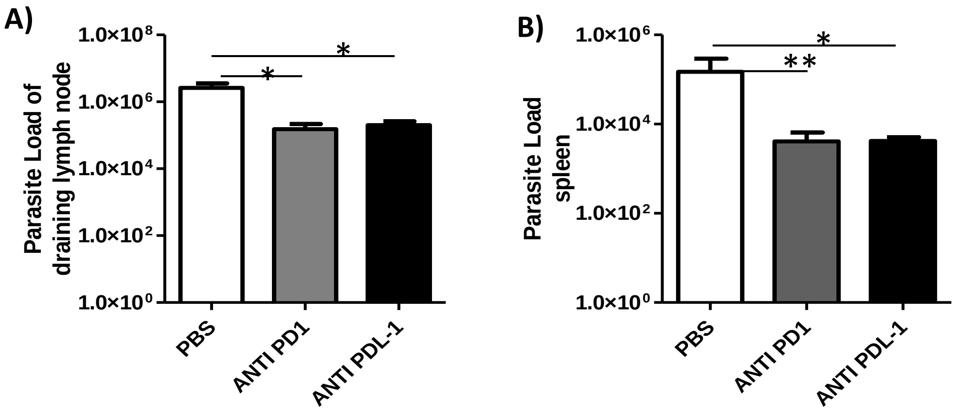

Suppl. Fig. 4: Effects of MoAb treatment on the specific anti-*Leishmania* immunoglobulins. (A) Specific IgM. (B) IgG. (C) IgG1. (D) IgG2a. Antibodies were detected in the serum (IgM, IgG and IgG1- diluted 1:500; IgG2a- diluted 1:40) of *L. amazonensis*-infected mice through ELISA using total *L. amazonensis* antigens (1 µg/well). Animals were treated for 49 days with antibodies administered twice a week intraperitoneally, starting at 7 days post- infection. Data ± SEM of individually mice (5 mice/group) are representative of two independent experiments producing the same result profile.

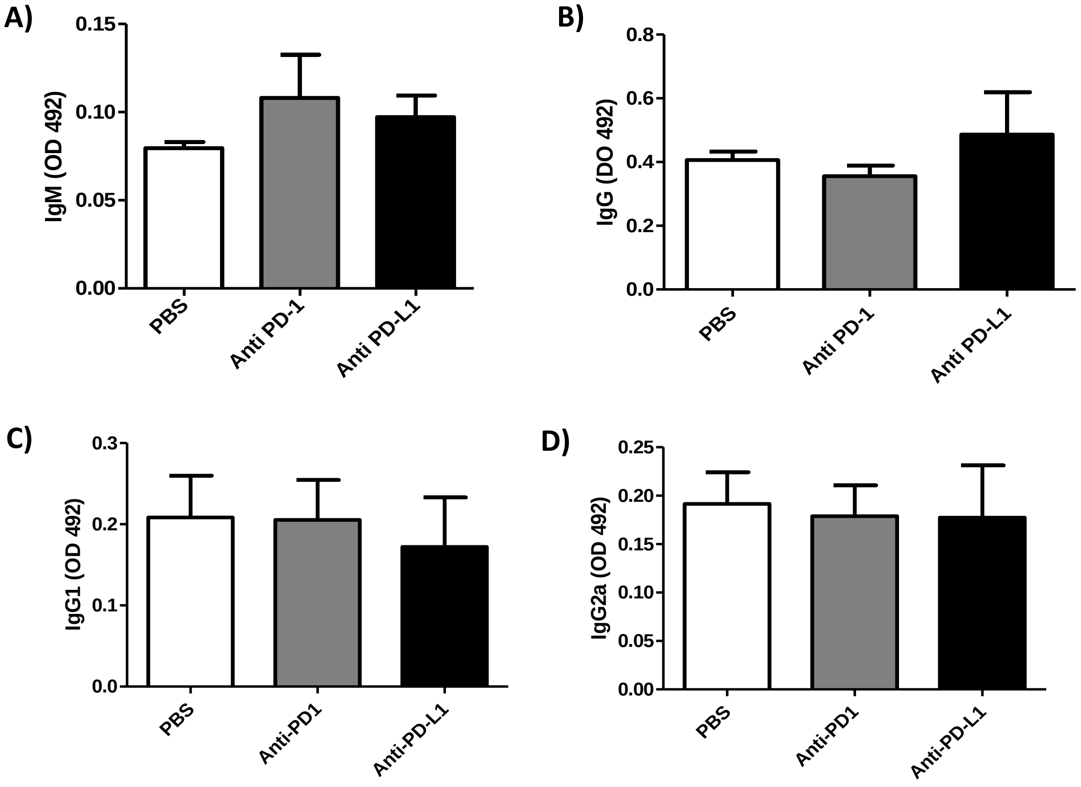

Suppl. Fig. 5: Treg cells in *L. amazonensis*-infected BALB/c mice. Mice were infected in the footpad with *L. amazonensis* promastigotes (2x10^6) and treated with anti-PD-1, anti-PD-L1 or anti-PD-L2, all at a 100 µg/dose administered twice per week intraperitoneally beginning at 7 days post-infection. Naive mice were used as control. Percentage of Treg cells. Data <u>+</u> SEM of individually mice (5 mice/group) are representative of two independent experiments producing the same result profile.

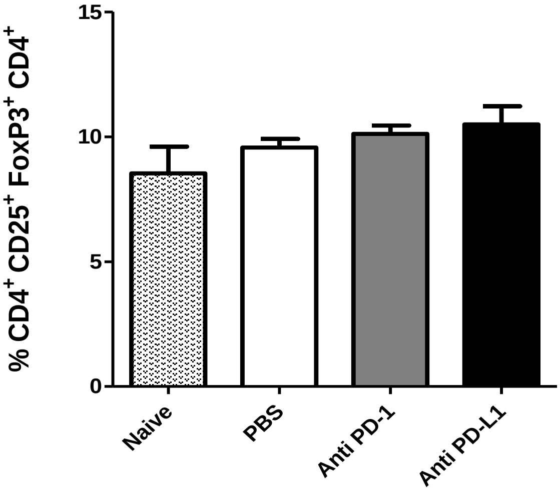

Suppl. Fig. 6: Analyse of IFN-ɤ production on CD3$^+$CD4$^-$ CD8$^-$. Lymphocytes were collected from the popliteal lymph node of an *L. amazonensis*-infected paw after approximately 2 months of treatment with anti-PD-1 or anti-PD-L1, administered twice a week intraperitoneally beginning after 7 days of infection.

(A) Percentage of CD4$^-$ CD8$^-$ T cells. (B) Number of CD4$^-$ CD8$^-$ T cells. (C) Dot plot of IFN-ɤ expression (APC-IFN-ɤ, SSC-A) . (D) Percentage of IFN-ɤ$^+$ CD4$^-$ CD8$^-$ T cells. (E) Number of IFN-ɤ$^+$ CD4$^-$ CD8$^-$ T cells. Naive = mice without infection and therapy, PBS = infected mice injected with PBS on treatment days, PD-1 = mice infected and treated with anti-PD-1 (100 µg/dose), PD-L1 = mice infected and treated with anti-PD-L1 (100 µg/dose). Data ± SEM of individually mice (5 mice/group) are representative of two independent experiments producing the same result profile.

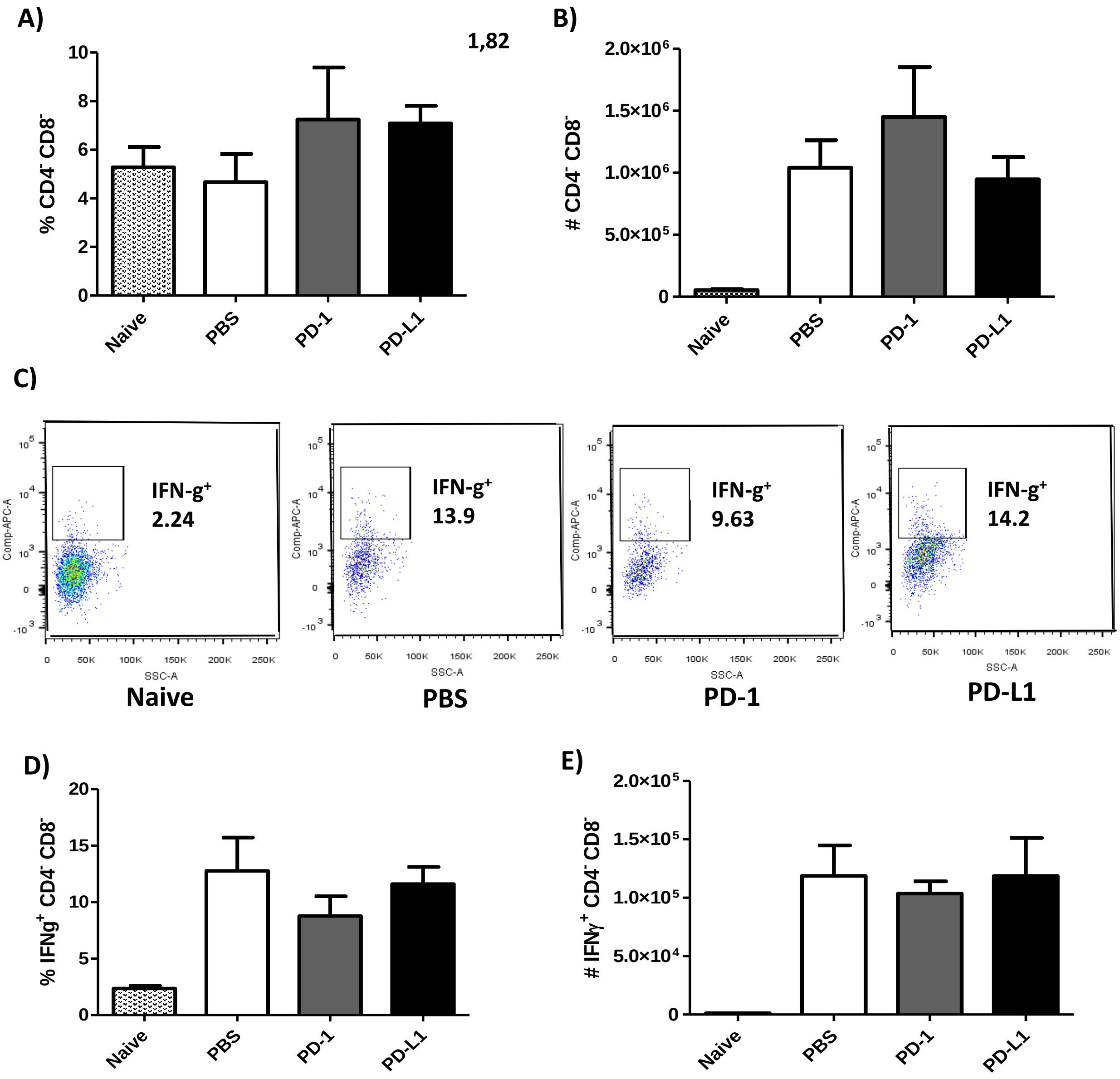

Suppl. Fig. 7: Analyse of IFN-γ^{+}PD-1^{+} CD4^{-} CD8^{-} T cells after anti-PD-1 and anti-PD-L1 MoAb treatment. Lymphocytes were collected from the popliteal lymph node of an *L. amazonensis*-infected paw after approximately 2 months of treatment with anti-PD-1 or anti-PD-L1, administered twice a week intraperitoneally beginning after 7 days of infection. (A) Dot plot of IFN-γ and PD-1 expression (APC-IFN-γ, PE-Cy7-PD-1). (B) Percentage of IFN-γ^{+} PD-1^{-} CD4^{-} CD8^{-} T cells. (C) Number of IFN-γ^{+}PD-1^{-}CD4^{-} CD8^{-} T cells. (D) Percentage of IFN-γ^{-}PD-1^{+} CD4^{-} CD8^{-} T cells. (E) Number of IFN-γ^{-} PD-1^{+} CD4^{-} CD8^{-} T cells. (F) Percentage of IFN-γ^{+}PD-1^{+} CD4^{-} CD8^{-} T cells. (G) Number of IFN-γ^{+}PD-1^{+} CD4^{-} CD8^{-} T cells. Naive = mice without infection and therapy, PBS = infected mice injected with PBS on treatment days, PD-1= mice infected and treated with anti-PD-1 (100 µg/dose), PD-L1= mice infected and treated with anti-PD-L1 (100 µg/dose). Data $\pm$ SEM of individually mice (5 mice/group) are representative of two independent experiments producing the same result profile.

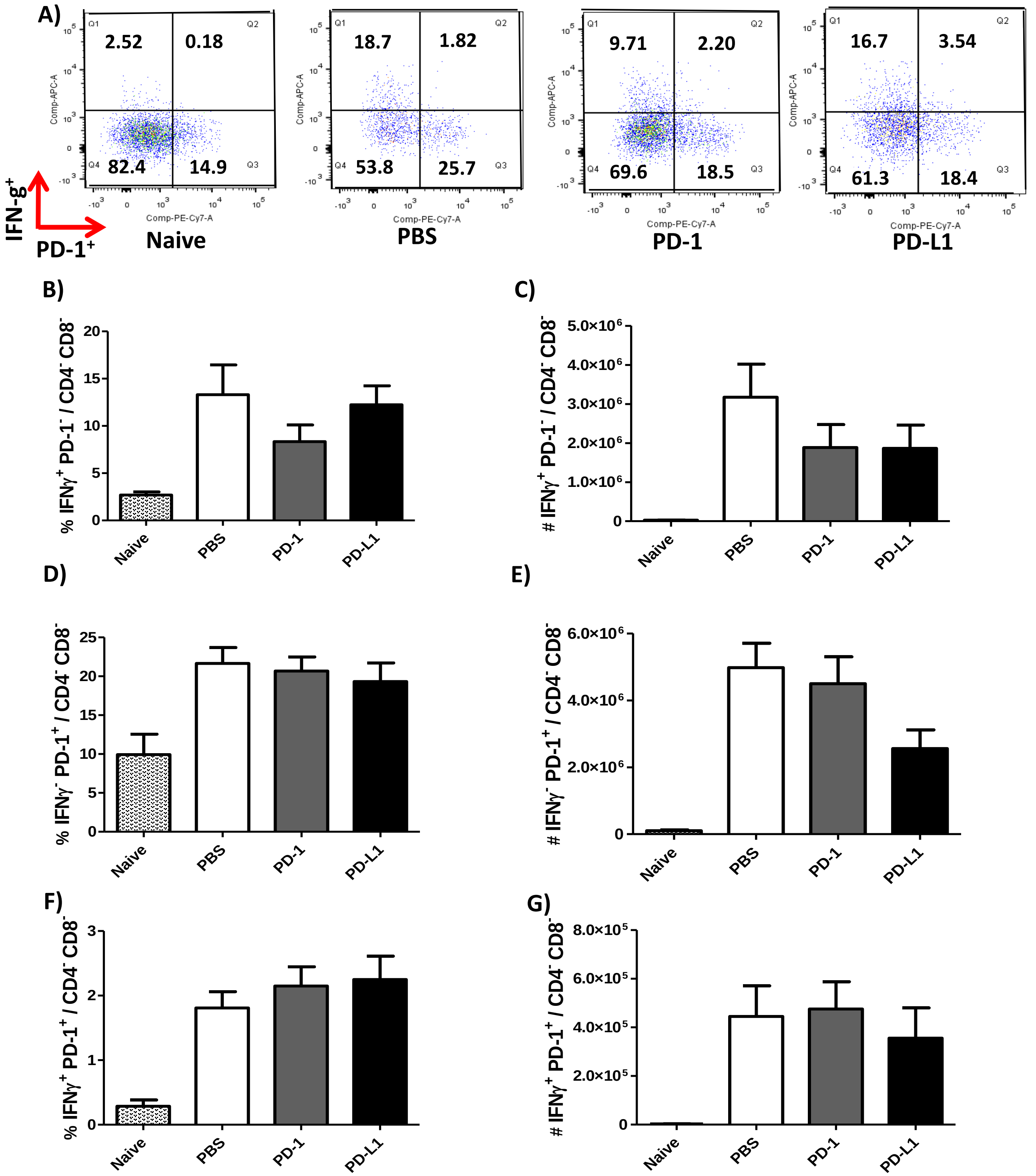

Suppl. Fig. 8: Strategy of gate of CD4 and CD8 T cells. (A) Lymphocytes-FSC x SSC. (B) Single cells- FSC-A x FSC-H. (c) $CD3^{+}$-APC cy7 x FSC-H. (D) $CD8^{+}CD3^{+}$-PerCP x $CD4^{+}CD3^{+}$-PE cy7 x DN- double negative. (E) IFN-ϒ- APC x PD-1- FITC . (F) IFN-ϒ- APC x FSC-A.

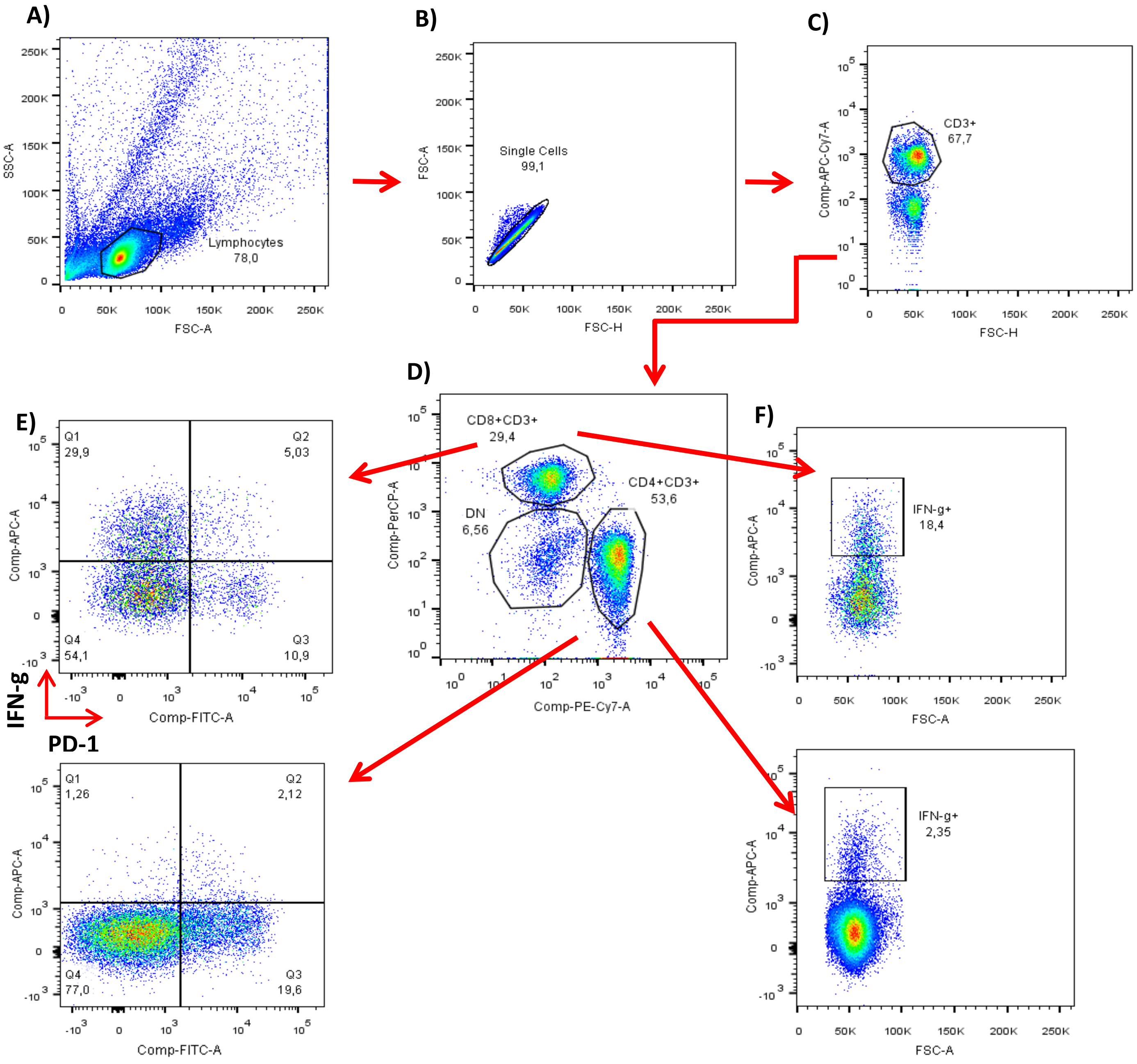

Suppl. Fig. 9: Strategy of gate of PD-L1$^+$ CD11c$^+$. (A) Cells- FSC x SSC. (B) Single cells- FSC-A x FSC-H. (c) CD11c$^+$- PerCP x SSC-H. (D) Histogram of PD-L1 expression. Red= negative of PD-L1, Blue= naïve and Orange= PD-L1$^+$ (APC-PD-L1).

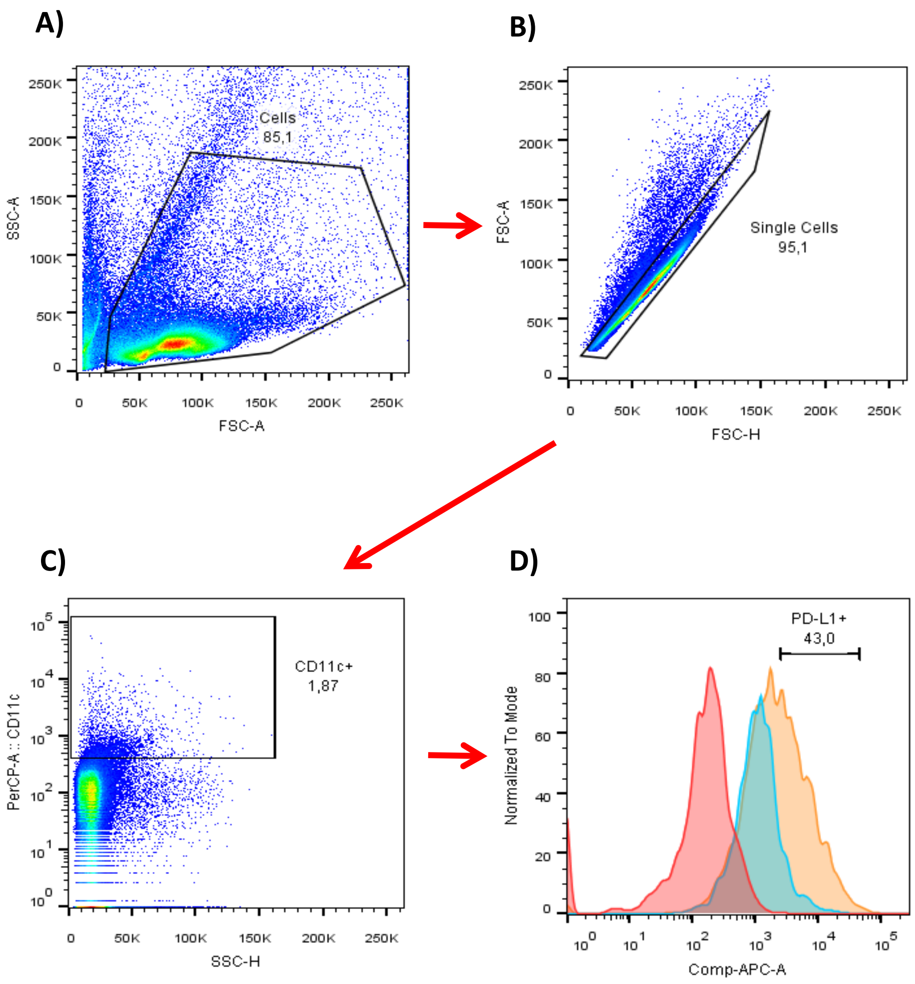

Supplement: Supplementary file 1 — Supplementary Figures [file 41598_2019_56336_MOESM1_ESM.pdf]
